# Supplementary material for: Within-individual changes reveal increasing social selectivity with age in rhesus macaques
Source: Proc Natl Acad Sci U S A. 2022 Nov 29;119(49):e2209180119. doi: 10.1073/pnas.2209180119 (PMC9894112; doi:10.1073/pnas.2209180119)
Supplement: Supplementary file 1 — Appendix 01(PDF) [file pnas.2209180119.sapp.pdf]

Supplementary Information for

Within-individual changes reveal increasing social selectivity with age in rhesus macaques

Erin R. Siracusa, Josué E. Negron-Del Valle, Daniel Phillips, Michael L. Platt, James P. Higham, Noah Snyder-Mackler, Lauren J. N. Brent

Correspondence to: Erin R. Siracusa

Email: erinsiracusa@gmail.com

This PDF file includes:

Supplementary Text
Tables S1 to S20
Figures S1 to S8
SI References

Supplementary Text

Separating within- from between-subject effects

In these analyses we were specifically interested in how social behavior changes across an individual's lifetime, that is, in the within-subject effect of age. To separate the within- from the between-subject age components we used a within-subjects centering approach (as per 1, 2). In a standard random effects linear regression, the estimated effect of a fixed term in the model includes both the within-subject effect and the between-subject effect, and is given by the following regression equation:

$$y_{ij} = \beta_0 + \beta_1 x_{ij} + u_{0j} + e_{0ij} \qquad \text{Equation 1}$$

where x_{ij} is the x value of measurement i from subject j. β_0 is the intercept of the regression equation and β_1 is the slope showing the effect of predictor variable, x, on the response variable, y, for the ith measurement of subject j. The terms u_{0j} and e_{0ij} denote the random intercept and residual variance, respectively.

To isolate a within-individual effect, we separated chronological age (x_{ij}) into two terms (see Figure 1D). The between-subject effect was obtained by taking the average age $\bar{x}$ of subject j, and was calculated by taking the mean age over all years that an individual was observed, resulting in one value of average age per individual $\bar{x}_j$ (Figure 1D). The within-subject effect (here called within-individual age) was calculated by subtracting average age from each age at which the individual was observed ($x_{ij} - \bar{x}_j$) resulting in many values per individual (one for each observation, centered around 0; Figure 1D). This within-subject centering model is given by the regression equation:

$$y_{ij} = \beta_0 + \beta_W(x_{ij} - \bar{x}_j) + \beta_B \bar{x}_j + u_{0j} + e_{0ij} \qquad \text{Equation 2}$$

Here β_W is the slope for the within-individual effect of age $(x_{ij} - \bar{x}_j)$ and β_B is the slope for the between-individual effect of average age $\bar{x}_j$. This β_B term therefore describes how the response variable (here some measure of social connectedness) is related to differences in age between individuals while β_W describes how the response variable changes with age across an individual's lifetime, relative to their mean age.

We modified the equation above to account for the possibility that age-related changes in social behavior could be driven by selective disappearance of individuals with high or low measures of social connectedness. For instance, less social individuals may be more likely to die young because of poorer access to resources (3-5). To test for the presence of selective disappearance we transformed the equation above to test whether the slopes β_B and β_W differed significantly from one another (1, 2). The new formulation of the equation is as follows:

$$y_{ij} = \beta_0 + \beta_W x_{ij} + (\beta_B - \beta_W)\, \bar{x}_j + u_{0j} + e_{0ij} \qquad \text{Equation 3}$$

Here β_W still represents the within-individual effect of age x_{ij} and is equivalent to β_W in Model 2 while the coefficient $(\beta_B - \beta_W)$ of average age $\bar{x}_j$ represents the difference between the between-individual and within-individual age terms. If this coefficient of average age is significantly

positive it would indicate that individuals with low social connectedness (or with low values of the given response variable) disappear selectively from the population because the slopes of the within- versus between-subject age differ significantly (1, 2).

Analyzing who aging females directed their approaches toward and received approaches from

We conducted a post-hoc analysis to assess whether older females directed approaches to, and received approaches from important partners (i.e., kin, strong, and stable partners). To do so we subset our data to only include the last year of data that we had for all subjects. For all subjects and their partners present in this "last-year" dataset we calculated the mean dyadic sociality index (DSI) using all previous years of data for the subject and their partner. For all subjects present in the last-year dataset we also assessed whether their partners were "stable" social partners (recorded as a categorical variable no/yes). Dyads were considered to be stable partners if they had a DSI > 0 for at least two consecutive years. Additional details of this methodological approach can be found in the Methods section of the main manuscript (see Predictions 4b and 4c). We predicted that individuals who were strongly connected to the subject earlier in life and who were stable partners with the subject earlier in life would be more likely to approach and be approached by the aging female than those individuals who previously had a weak or unstable social relationship with the subject.

All statistical analyses were conducted at the level of the dyad. We fitted whether or not individuals approached or were approached by the aging female (coded as 0/1) as the response variables of interest in separate models and used a Bernoulli error distribution (logit link). For the two models looking at whether approaches were predicted by the strength of relationship between the female and her partner, the predictor variable of interest was the mean DSI for the focal individual and their partner – calculated based on all previous years of interaction. This was included in the model as a continuous fixed effect. For the two models looking at whether approaches were predicted by the stability of the relationship between the female and her partner, the predictor of interest was whether or not the focal individual and partner were stable social partners in previous years (no/yes). We included this in the model as a categorical fixed effect. We also included the partner's age (continuous), the partner's rank (categorical) and whether or not the partner was kin or non-kin (categorical) as fixed effects in all models (see Tables S17-S20). The inclusion of a fixed effect for kin or non-kin allowed us to assess whether females were preferentially approaching or being approached by related individuals. In each model we checked for an interaction between the predictor of interest (mean DSI and partner stability) and focal individual age to assess whether the likelihood of approaching or being approached by a strong or stable partner was dependent on a female's age. We removed the interaction term from the model when it was not significant. We included group and year as random effects to account for variation in approaches that might be due to differences between groups or years. Individual ID and partner ID were included as random effects in a multi-membership grouping term (6). This multi-membership grouping term accounts for the inherent multilevel structure of the data and allows each sample (dyad) to belong to more than one individual in a random effect at the same time.

At a dyadic level, older female macaques were more likely to approach (β = 0.16; 95% CI = [0.14, 0.18]; Table S17; Figure S7 *A* and *B*) and be approached by (β = 0.14; 95% CI = [0.13,

0.17]; Table S18; Figure S7 *C* and *D*) partners to whom they had previously been strongly connected. Females were also more likely to approach individuals with whom they had a stable social relationship earlier in life, and this effect was strongest in the oldest females (partner stableY*age: $\beta = 0.06$; 95% CI = [0.02, 0.11]; Table S19 Figure S8 *A* and *B*). Similarly, females were more likely to be approached by stable partners ($\beta = 1.16$; 95% CI = [0.92, 1.39]; Table S20; Figure S8 *C* and *D*). We also found evidence in all models that females were more like to approach and be approached by kin (see Tables S17-S20).

Table S1. Fixed and random effects from models looking at the effects of age on number of grooming partners (grooming degree). Model A is the within-individual centering model (based on Equation 2, see Supplementary Text), and Model B is the reformulation of this model (based on Equation 3, see Supplementary Text) to test for selective disappearance. Bolded terms indicate fixed effects where the 95% credible intervals did not overlap zero, providing evidence that those effects were significantly different from zero. Given that the 95% credible intervals for the average age term in Model B overlapped zero there was no evidence for selective disappearance.

We ran both models with the following weakly informative prior means and standard deviations (μ, σ): intercept (2, 1), within-age (0, 0.5), average-age (0, 0.5), rankL (0, 0.5), rankM (0, 0.5).

Model	Effect	Group	Term	Estimate	Lower 95% CI	Upper 95% CI
**Model A**	Fixed Effects		intercept	2.01	1.52	2.52
			**within-age**	**-0.06**	**-0.12**	**-0.01**
			**average-age**	**-0.04**	**-0.06**	**-0.02**
			**rankL**	**-0.42**	**-0.56**	**-0.28**
			**rankM**	**-0.27**	**-0.4**	**-0.12**
	Random Effects	Group	sd(intercept)	0.38	0.15	0.91
		Year	sd(intercept)	0.32	0.17	0.62
		Individual.ID	sd(intercept)	0.22	0.14	0.30
			sd(within-age)	0.03	0.00	0.08
			cor(intercept,within-age)	0.10	-0.92	0.95
**Model B**	Fixed Effects		intercept	2.01	1.51	2.54
			**age**	**-0.06**	**-0.11**	**-0.01**
			average-age	0.02	-0.04	0.08
			**rankL**	**-0.42**	**-0.55**	**-0.29**
			**rankM**	**-0.27**	**-0.40**	**-0.13**
	Random Effects	Group	sd(intercept)	0.39	0.15	0.92
		Year	sd(intercept)	0.32	0.17	0.63
		Individual.ID	sd(intercept)	0.22	0.14	0.29

Table S2. Fixed and random effects from models looking at the effects of age on number of proximity partners (proximity degree). Model A is the within-individual centering model (based on Equation 2, see Supplementary Text), and Model B is the reformulation of this model (based on Equation 3, see Supplementary Text) to test for selective disappearance. Bolded terms indicate fixed effects where the 95% credible intervals did not overlap zero, providing evidence that those effects were significantly different from zero. Given that the 95% credible intervals for the average age term in Model B overlapped zero there was no evidence for selective disappearance.

We ran both models with the following weakly informative prior means and standard deviations (μ, σ): intercept (5, 2), within-age (0, 0.5), average-age (0, 0.5), rankL (0, 0.5), rankM (0, 0.5), within-age:rankL (0, 0.5), within-age:rankM (0, 0.5).

Model	Effect	Group	Term	Estimate	Lower 95% CI	Upper 95% CI
**Model A**	Fixed Effects		intercept	2.85	2.17	3.61
			within-age	-0.01	-0.08	0.05
			**average-age**	**-0.03**	**-0.04**	**-0.01**
			rankM	-0.10	-0.21	0.01
			**rankL**	**-0.29**	**-0.4**	**-0.18**
			**within-age:rankL**	**-0.07**	**-0.13**	**-0.01**
			within-age:rankM	-0.03	-0.09	0.03
	Random Effects	Group	sd(intercept)	0.61	0.29	1.35
		Year	sd(intercept)	0.57	0.31	1.08
		Individual.ID	sd(intercept)	0.26	0.22	0.31
			sd(within-age)	0.05	0.02	0.08
			cor(intercept,within-age)	0.67	0.17	0.98
**Model B**	Fixed Effects		intercept	2.56	1.83	3.36
			age	-0.03	-0.08	0.01
			average-age	0.03	-0.02	0.08
			rankM	0.24	-0.1	0.57
			rankL	0.10	-0.24	0.44
			**age:ordinal.rankL**	**-0.03**	**-0.05**	**-0.01**
			age:ordinal.rankM	-0.03	-0.05	0.00
	Random Effects	Group	sd(intercept)	0.64	0.30	1.39
		Year	sd(intercept)	0.57	0.32	1.10
		Individual.ID	sd(intercept)	0.26	0.22	0.31

Figure S1. Effects of age on within-individual changes in (A) the number of grooming partners and (B) the number of proximity partners (results shown on chronological age scale based on Model B, Tables S1-2). Points represent raw data. Shaded gray ribbons indicate 95% confidence intervals around the predicted values.

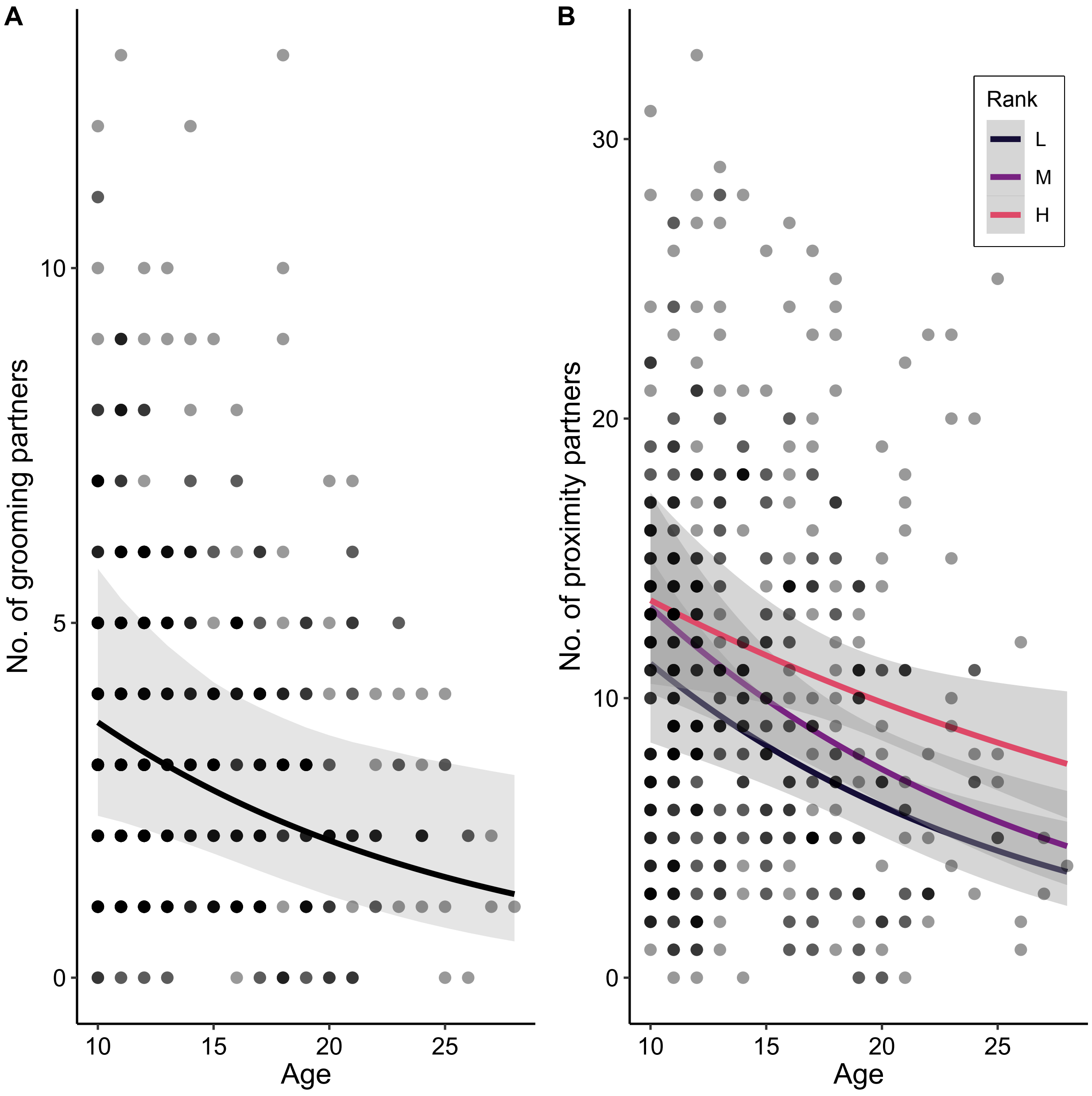

Table S3. Fixed and random effects from models looking at the effects of age on number of partners that a focal individual approached (approach outdegree). Model A is the within-individual centering model (based on Equation 2, see Supplementary Text), and Model B is the reformulation of this model (based on Equation 3, see Supplementary Text) to test for selective disappearance. Bolded terms indicate fixed effects where the 95% credible intervals did not overlap zero, providing evidence that those effects were significantly different from zero. Given that the 95% credible intervals for the average age term in Model B overlapped zero there was no evidence for selective disappearance.

We ran both models with the following weakly informative prior means and standard deviations (μ, σ): intercept (0, 3), within-age (0, 0.5), average-age (0, 0.5), rankL (0, 0.5), rankM (0, 0.5), within-age:rankL (0, 0.5), within-age:rankM (0, 0.5).

Model	Effect	Group	Term	Estimate	Lower 95% CI	Upper 95% CI
**Model A**	Fixed Effects		intercept	2.73	2.08	3.37
			within-age	-0.02	-0.08	0.04
			**average-age**	**-0.03**	**-0.04**	**-0.01**
			**rankL**	**-0.44**	**-0.55**	**-0.32**
			rankM	-0.09	-0.2	0.02
			**within-age:rankL**	**-0.08**	**-0.14**	**-0.01**
			within-age:rankM	-0.02	-0.08	0.04
	Random Effects	Group	sd(intercept)	0.52	0.25	1.14
		Year	sd(intercept)	0.56	0.31	1.05
		Individual.ID	sd(intercept)	0.28	0.24	0.34
			sd(within-age)	0.06	0.03	0.08
			cor(intercept,within-age)	0.87	0.56	1.00
**Model B**	Fixed Effects		intercept	2.54	1.87	3.21
			age	-0.03	-0.07	0.01
			average-age	0.02	-0.03	0.07
			rankL	-0.07	-0.42	0.28
			rankM	0.11	-0.21	0.44
			**age:ordinal.rankL**	**-0.03**	**-0.05**	**-0.01**
			age:ordinal.rankM	-0.02	-0.04	0.00
	Random Effects	Group	sd(intercept)	0.52	0.25	1.13
		Year	sd(intercept)	0.57	0.31	1.07
		Individual.ID	sd(intercept)	0.28	0.23	0.33

Table S4. Fixed and random effects from models looking at the effects of age on number of partners that a focal individual was approached by (approach indegree). Model A is the within-individual centering model (based on Equation 2, see Supplementary Text), and Model B is the reformulation of this model (based on Equation 3, see Supplementary Text) to test for selective disappearance. Bolded terms indicate fixed effects where the 95% credible intervals did not overlap zero, providing evidence that those effects were significantly different from zero. Given that the 95% credible intervals for the average age term in Model B overlapped zero there was no evidence for selective disappearance.

We ran both models with the following weakly informative prior means and standard deviations (μ, σ): intercept (0, 3), within-age (0, 0.5), average-age (0, 0.5), rankL (0, 0.5), rankM (0, 0.5).

Model	Effect	Group	Term	Estimate	Lower 95% CI	Upper 95% CI
**Model A**	Fixed Effects		intercept	2.60	1.97	3.21
			within-age	-0.02	-0.07	0.02
			**average-age**	**-0.03**	**-0.04**	**-0.01**
			rankL	-0.01	-0.12	0.10
			rankM	0.05	-0.05	0.15
	Random Effects	Group	sd(intercept)	0.48	0.22	1.07
		Year	sd(intercept)	0.55	0.30	1.04
		Individual.ID	sd(intercept)	0.27	0.22	0.31
			sd(within-age)	0.04	0.01	0.07
			cor(intercept,within-age)	0.70	0.16	0.98
**Model B**	Fixed Effects		intercept	2.59	1.98	3.19
			age	-0.02	-0.06	0.02
			average-age	-0.01	-0.06	0.04
			rankL	-0.04	-0.15	0.06
			rankM	0.03	-0.07	0.12
	Random Effects	Group	sd(intercept)	0.48	0.23	1.06
		Year	sd(intercept)	0.54	0.3	1.03
		Individual.ID	sd(intercept)	0.26	0.22	0.31

Figure S2. Effects of age on within-individual changes in (A) the number of partners that a focal individual approached and (B) the number of partners that a focal individual was approached by (results shown on chronological age scale based on Model B, Tables S3-4). Points represent raw data. Shaded gray ribbons indicate 95% confidence intervals around the predicted values.

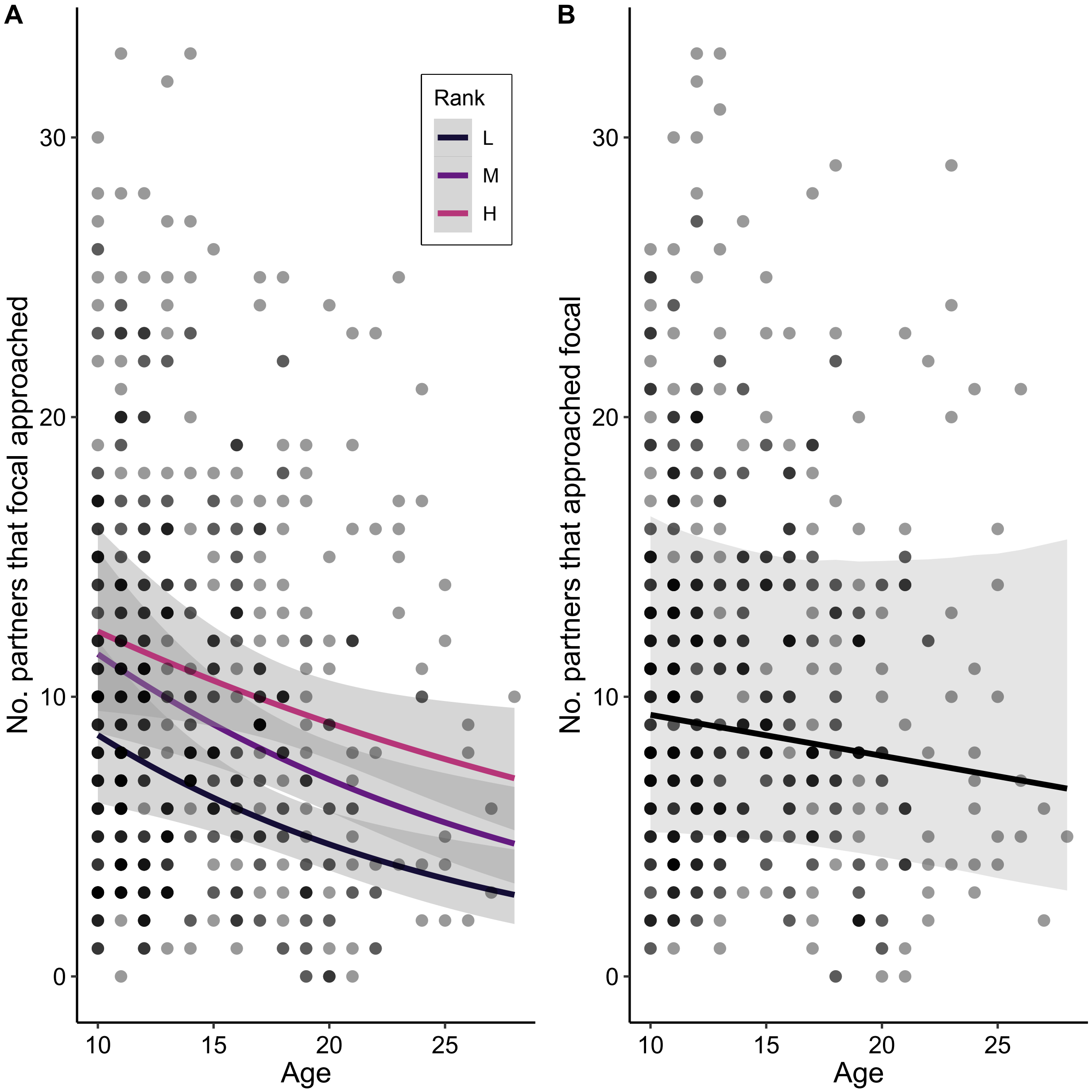

Table S5. Fixed and random effects from models looking at the effects of partner deaths in year t-1 on number of grooming partners in year t. Bolded terms indicate fixed effects where the 95% credible intervals did not overlap zero, providing evidence that those effects were significantly different from zero.

We ran the model with the following weakly informative prior means and standard deviations (μ, σ): intercept (0, 1), no.partner.deaths (0, 0.5), groupR (0, 0.5), groupV (0, 0.5).

Effect	Group	Term	Estimate	Lower 95% CI	Upper 95% CI
**Fixed Effects**		intercept	1.11	0.75	1.42
		no.partner.deaths	0.06	-0.04	0.15
		groupR	-0.15	-0.39	0.08
		**groupV**	**-0.36**	**-0.63**	**-0.1**
**Random Effects**	Year	sd(intercept)	0.4	0.2	0.83
	Individual.ID	sd(intercept)	0.3	0.22	0.4

Table S6. Fixed and random effects from models looking at the effects of partner deaths in year t-1 on number of proximity partners in year t. Bolded terms indicate fixed effects where the 95% credible intervals did not overlap zero, providing evidence that those effects were significantly different from zero.

We ran the model with the following weakly informative prior means and standard deviations (μ, σ): intercept (0, 1), no.partner.deaths (0, 0.5), groupR (0, 0.5), groupV (0, 0.5).

Effect	Group	Term	Estimate	Lower 95% CI	Upper 95% CI
**Fixed Effects**		intercept	1.92	1.25	2.41
		**no.partner.deaths**	**0.12**	**0.05**	**0.18**
		groupR	0.14	-0.05	0.32
		**groupV**	**-0.68**	**-0.92**	**-0.45**
**Random Effects**	Year	sd(intercept)	0.69	0.36	1.43
	Individual.ID	sd(intercept)	0.34	0.27	0.41

Table S7. Fixed and random effects from models looking at the effects of age on the amount of time spent giving grooming (grooming outstrength). Model A is the within-individual centering model (based on Equation 2, see Supplementary Text), and Model B is the reformulation of this model (based on Equation 3, see Supplementary Text) to test for selective disappearance. Bolded terms indicate fixed effects where the 95% credible intervals did not overlap zero, providing evidence that those effects were significantly different from zero. Given that the 95% credible intervals for the average age term in Model B overlapped zero there was no evidence for selective disappearance.

We ran both models with the following weakly informative prior means and standard deviations (μ, σ): intercept (0, 5), within-age (0, 0.5), average-age (0, 0.5), rankL (0, 0.5), rankM (0, 0.5).

Model	Effect	Group	Term	Estimate	Lower 95% CI	Upper 95% CI
**Model A**	Fixed Effects		intercept	-3.63	-4.21	-3.06
			within-age	-0.05	-0.12	0.03
			average-age	-0.03	-0.05	0.00
			rankL	-0.11	-0.30	0.08
			rankM	-0.12	-0.31	0.08
	Random Effects	Group	sd(intercept)	0.27	0.05	0.72
		Year	sd(intercept)	0.39	0.18	0.80
		Individual.ID	sd(intercept)	0.29	0.18	0.39
			sd(within-age)	0.07	0.01	0.15
			cor(intercept,within-age)	-0.57	-0.98	0.24
**Model B**	Fixed Effects		intercept	-3.62	-4.16	-3.06
			age	-0.04	-0.11	0.03
			average-age	0.02	-0.06	0.10
			rankL	-0.12	-0.31	0.07
			rankM	-0.12	-0.32	0.07
	Random Effects	Group	sd(intercept)	0.25	0.05	0.65
		Year	sd(intercept)	0.38	0.18	0.77
		Individual.ID	sd(intercept)	0.28	0.17	0.39

Table S8. Fixed and random effects from models looking at the effects of age on the amount of time spent receiving grooming (grooming instrength). Model A is the within-individual centering model (based on Equation 2, see Supplementary Text), and Model B is the reformulation of this model (based on Equation 3, see Supplementary Text) to test for selective disappearance. Bolded terms indicate fixed effects where the 95% credible intervals did not overlap zero, providing evidence that those effects were significantly different from zero. Given that the 95% credible intervals for the average age term in Model B overlapped zero there was no evidence for selective disappearance.

We ran both models with the following weakly informative prior means and standard deviations (μ, σ): intercept (0, 5), within-age (0, 0.5), average-age (0, 0.5), rankL (0, 0.5), rankM (0, 0.5).

Model	Effect	Group	Term	Estimate	Lower 95% CI	Upper 95% CI
**Model A**	Fixed Effects		intercept	-3.56	-4.17	-2.95
			within-age	0.02	-0.05	0.09
			average-age	-0.01	-0.03	0.01
			**rankL**	**-0.38**	**-0.54**	**-0.21**
			**rankM**	**-0.34**	**-0.52**	**-0.16**
	Random Effects	Group	sd(intercept)	0.45	0.18	1.05
		Year	sd(intercept)	0.37	0.17	0.75
		Individual.ID	sd(intercept)	0.13	0.01	0.26
			sd(within-age)	0.05	0.00	0.12
			cor(intercept,within-age)	-0.23	-0.97	0.88
**Model B**	Fixed Effects		intercept	-3.55	-4.16	-2.93
			age	0.02	-0.04	0.09
			average-age	-0.03	-0.11	0.04
			**rankL**	**-0.38**	**-0.54**	**-0.21**
			**rankM**	**-0.35**	**-0.52**	**-0.17**
	Random Effects	Group	sd(intercept)	0.45	0.18	1.07
		Year	sd(intercept)	0.37	0.17	0.75
		Individual.ID	sd(intercept)	0.13	0.01	0.26

Table S9. Fixed and random effects from models looking at the effects of age on the amount of time spent in proximity to other females (proximity strength). Model A is the within-individual centering model (based on Equation 2, see Supplementary Text), and Model B is the reformulation of this model (based on Equation 3, see Supplementary Text) to test for selective disappearance. Bolded terms indicate fixed effects where the 95% credible intervals did not overlap zero, providing evidence that those effects were significantly different from zero. Given that the 95% credible intervals for the average age term in Model B overlapped zero there was no evidence for selective disappearance.

We ran both models with the following weakly informative prior means and standard deviations (μ, σ): intercept (0, 5), within-age (0, 0.5), average-age (0, 0.5), rankL (0, 0.5), rankM (0, 0.5).

Model	Effect	Group	Term	Estimate	Lower 95% CI	Upper 95% CI
**Model A**	Fixed Effects		intercept	-1.21	-2.22	-0.20
			within-age	-0.02	-0.08	0.05
			average-age	-0.02	-0.04	0.00
			**rankL**	**-0.53**	**-0.68**	**-0.37**
			**rankM**	**-0.32**	**-0.47**	**-0.17**
	Random Effects	Group	sd(intercept)	0.81	0.39	1.74
		Year	sd(intercept)	0.86	0.48	1.61
		Individual.ID	sd(intercept)	0.34	0.28	0.41
			sd(witihin-age)	0.08	0.01	0.20
			cor(intercept,within-age)	-0.03	-0.81	0.60
**Model B**	Fixed Effects		intercept	-1.21	-2.23	-0.18
			age	-0.02	-0.08	0.04
			average-age	0.00	-0.07	0.08
			**rankL**	**-0.51**	**-0.66**	**-0.37**
			**rankM**	**-0.35**	**-0.49**	**-0.21**
	Random Effects	Group	sd(intercept)	0.81	0.40	1.76
		Year	sd(intercept)	0.88	0.49	1.65
		Individual.ID	sd(intercept)	0.34	0.27	0.40

Figure S3. Effects of age on within-individual changes in (A) amount of grooming given, (B) amount of grooming received, and (C) amount of time spent in proximity to other females (results shown on chronological age scale based on Model B, Tables S7-9). Points represent raw data. Shaded gray ribbons indicate 95% confidence intervals around the predicted values.

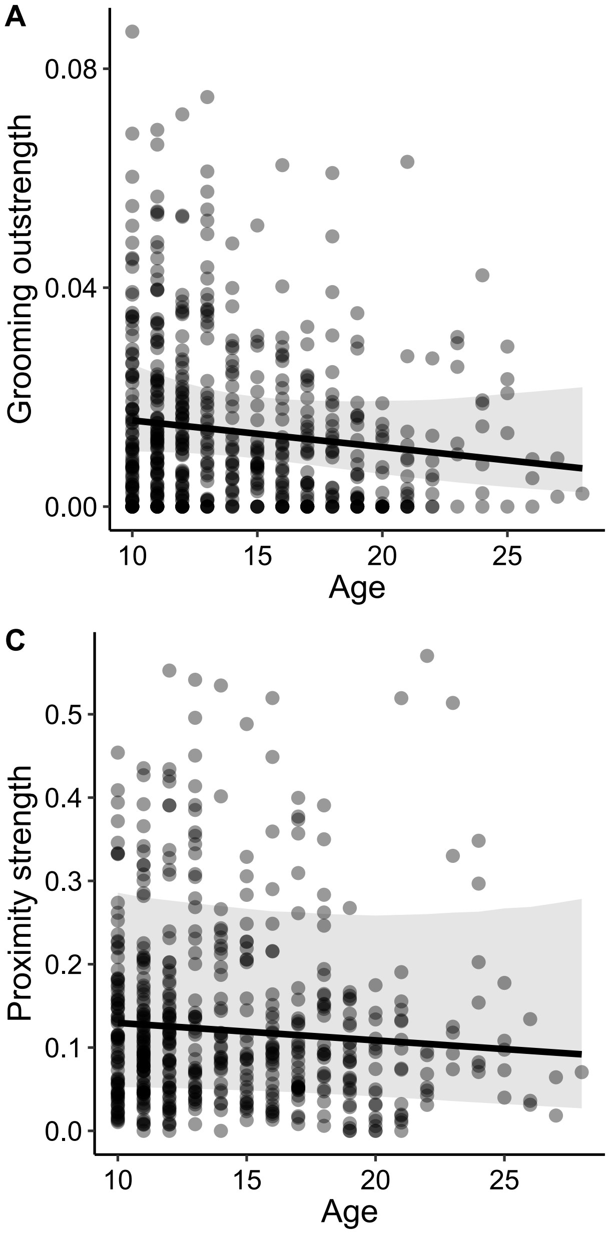

Table S10. Fixed and random effects from models looking at the effects of age on the proportion of kin partners. Model A is the within-individual centering model (based on Equation 2, see Supplementary Text), and Model B is the reformulation of this model (based on Equation 3, see Supplementary Text) to test for selective disappearance. Bolded terms indicate fixed effects where the 95% credible intervals did not overlap zero, providing evidence that those effects were significantly different from zero. Given that the 95% credible intervals for the average age term in Model B overlapped zero there was no evidence for selective disappearance.

We ran both models with the following weakly informative prior means and standard deviations (μ, σ): intercept (0, 5), within-age (0, 0.5), average-age (0, 0.5), rankL (0, 0.5), rankM (0, 0.5).

Model	Effect	Group	Term	Estimate	Lower 95% CI	Upper 95% CI
**Model A**	Fixed Effects		intercept	0.06	-0.72	0.80
			**within-age**	**0.12**	**0.03**	**0.19**
			**average-age**	**0.05**	**0.03**	**0.08**
			**rankL**	**0.24**	**0.01**	**0.46**
			rankM	0.03	-0.19	0.25
	Random Effects	Group	sd(intercept)	0.65	0.28	1.44
		Year	sd(intercept)	0.21	0.06	0.50
		Individual.ID	sd(intercept)	0.28	0.14	0.41
			sd(within-age)	0.04	0.00	0.10
			cor(intercept,within-age)	0.32	-0.85	0.98
**Model B**	Fixed Effects		intercept	0.07	-0.69	0.84
			**age**	**0.12**	**0.03**	**0.19**
			average-age	-0.06	-0.15	0.04
			**rankL**	**0.24**	**0.02**	**0.46**
			rankM	0.04	-0.18	0.25
	Random Effects	Group	sd(intercept)	0.67	0.29	1.52
		Year	sd(intercept)	0.21	0.06	0.49
		Individual.ID	sd(intercept)	0.27	0.12	0.40

Figure S4. Effects of age on within-individual changes in the proportion of kin partners (results shown on chronological age scale based on Model B, Table S10). Points represent raw data. Shaded gray ribbons indicate 95% confidence intervals around the predicted values.

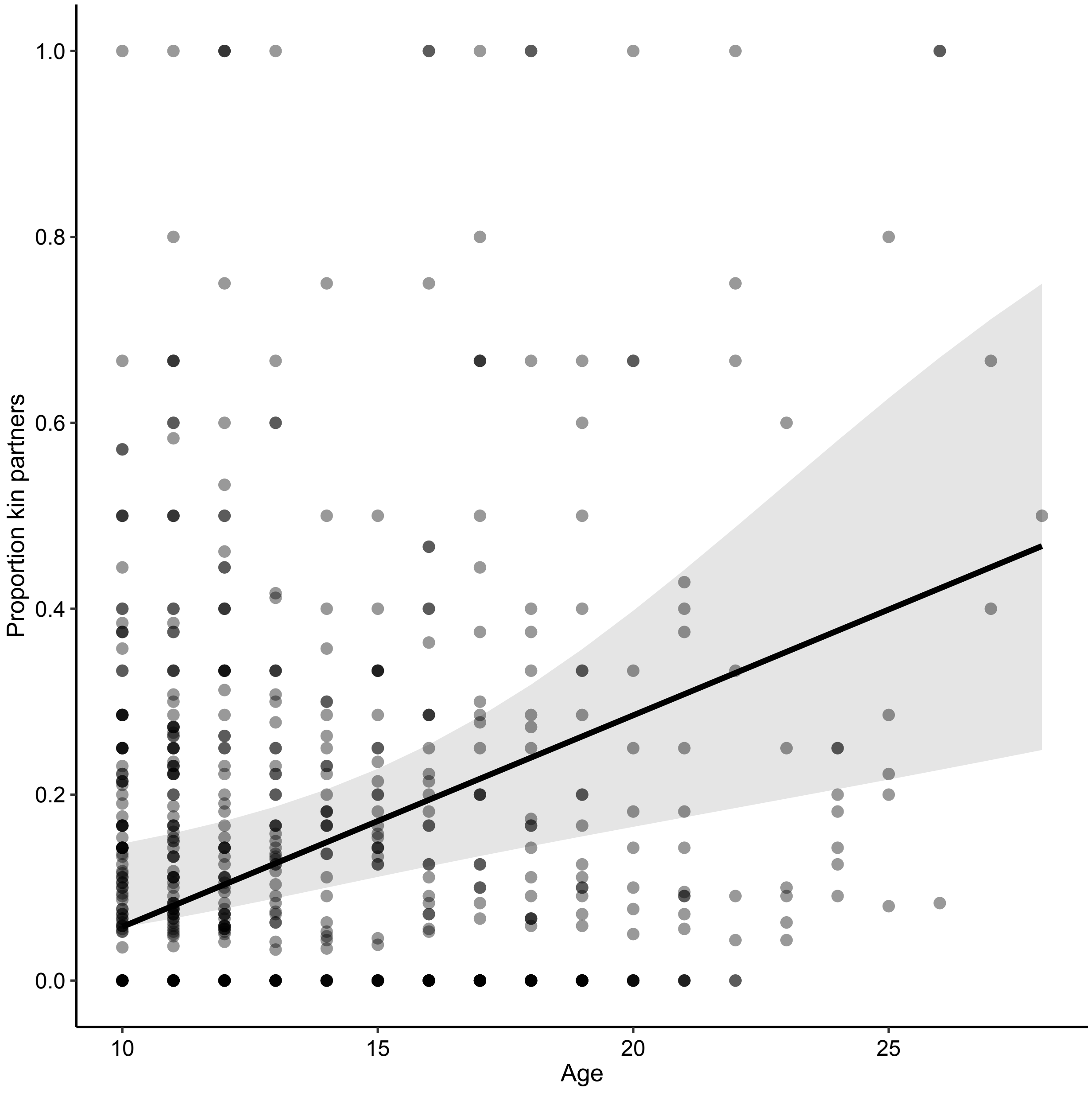

Table S11. Fixed and random effects from models looking at whether the mean dyadic sociality index (DSI) between a female and her partner predicts the probability of those individuals being partners later in the female's life. Bolded terms indicate fixed effects where the 95% credible intervals did not overlap zero, providing evidence that those effects were significantly different from zero.

We ran the model with the following weakly informative prior means and standard deviations (μ, σ): intercept (0, 1), mean DSI (0, 1), partner age (0, 1), partner rankL (0, 1), partner rankM (0, 1), partner relatedness_nonkin (0, 1), mean DSI:age (0, 1).

Effect	Group	Term	Estimate	Lower 95% CI	Upper 95% CI
**Fixed Effects**		intercept	0.67	-0.51	2.05
		mean DSI	0.05	-0.04	0.15
		partner age	-0.02	-0.04	0.00
		**partner rankL**	**-0.42**	**-0.60**	**-0.24**
		partner rankM	-0.12	-0.31	0.07
		**partner relatedness_nonkin**	**-1.07**	**-1.25**	**-0.88**
		**age**	**-0.04**	**-0.06**	**-0.01**
		**mean DSI:age**	**0.01**	**0.01**	**0.02**
**Random Effects**	Group	sd(intercept)	1.31	0.58	2.95
	Year	sd(intercept)	0.91	0.44	1.84
	Multi-membership	sd(intercept)	0.87	0.74	1.01

Table S12. Fixed and random effects from models looking at whether the stability between a female and her partner (i.e., being partners for at least two consecutive years) predicts the probability of those individuals being partners later in the female's life. Bolded terms indicate fixed effects where the 95% credible intervals did not overlap zero, providing evidence that those effects were significantly different from zero.

We ran the model with the following weakly informative prior means and standard deviations (μ, σ): intercept (0, 0.5), partner stableY (0, 1), partner age (0, 1), partner rankL (0, 1), partner rankM (0, 1), partner relatedness_nonkin (0, 1), groupV (0, 1), groupKK (0, 1).

Effect	Group	Term	Estimate	Lower 95% CI	Upper 95% CI
Fixed Effects		intercept	0.70	-0.41	1.93
		**partner stableY**	**1.54**	**1.31**	**1.77**
		partner age	-0.03	-0.05	0.00
		**partner rankL**	**-0.37**	**-0.62**	**-0.12**
		partner rankM	-0.08	-0.34	0.18
		**partner relatedness_nonkin**	**-1.59**	**-1.83**	**-1.36**
		**groupV**	**-0.47**	**-0.92**	**-0.01**
		**groupKK**	**1.82**	**1.43**	**2.21**
Random Effects	Year	sd(intercept)	1.57	0.46	3.51
	Multi-membership	sd(intercept)	0.86	0.68	1.05

Table S13. Fixed and random effects from models looking at whether the mean grooming rate (i.e., grooming strength) between a female and her partner predicts the probability of those individuals being grooming partners later in the female's life. Bolded terms indicate fixed effects where the 95% credible intervals did not overlap zero, providing evidence that those effects were significantly different from zero.

We ran the model with the following weakly informative prior means and standard deviations (μ, σ): intercept (0, 1), mean groom rate (0, 1), partner age (0, 1), partner rankL (0, 1), partner rankM (0, 1), partner relatedness_nonkin (0, 1), mean groom rate:age (0, 1).

Effect	Group	Term	Estimate	Lower 95% CI	Upper 95% CI
Fixed Effects		intercept	0.56	-0.75	2.74
		mean groom rate	0.53	-1.42	2.48
		**partner age**	**-0.04**	**-0.07**	**-0.01**
		**partner rankL**	**-0.43**	**-0.69**	**-0.16**
		**partner rankM**	**-0.3**	**-0.58**	**-0.02**
		**partner relatedness_nonkin**	**-1.86**	**-2.09**	**-1.63**
		**age**	**-0.05**	**-0.08**	**-0.02**
		**mean groom rate:age**	**7.43**	**6.28**	**8.59**
Random Effects	Group	sd(intercept)	1.09	0.24	3.47
	Year	sd(intercept)	0.71	0.24	2.02
	Multi-membership	sd(intercept)	0.95	0.74	1.16

Table S14. Fixed and random effects from models looking at whether the mean proximity rate (i.e., proximity strength) between a female and her partner predicts the probability of those individuals being proximity partners later in the female's life. Bolded terms indicate fixed effects where the 95% credible intervals did not overlap zero, providing evidence that those effects were significantly different from zero.

We ran the model with the following weakly informative prior means and standard deviations (μ, σ): intercept (0, 1), mean proximity rate (0, 1), partner age (0, 1), partner rankL (0, 1), partner rankM (0, 1), partner relatedness_nonkin (0, 1), mean proximity rate:age (0, 1).

Effect	Group	Term	Estimate	Lower 95% CI	Upper 95% CI
**Fixed Effects**		intercept	0.65	-0.57	2.11
		mean proximity rate	0.42	-1.52	2.38
		partner age	-0.02	-0.04	0.00
		**partner rankL**	**-0.44**	**-0.62**	**-0.25**
		partner rankM	-0.14	-0.34	0.05
		**partner relatedness_nonkin**	**-1.16**	**-1.35**	**-0.97**
		**age**	**-0.03**	**-0.06**	**-0.01**
		**mean prox rate:age**	**5.27**	**4.57**	**5.97**
**Random Effects**	Group	sd(intercept)	1.26	0.55	2.90
	Year	sd(intercept)	0.91	0.43	1.90
	Multi-membership	sd(intercept)	0.91	0.77	1.06

Figure S5. Effects of the strength of the (A & B) grooming relationship and (C & D) proximity relationship between a female and her partner on the probability of that individual being chosen as a grooming or proximity partner, respectively, by the female in later life. Points represent raw data. Shaded gray ribbons indicate 95% confidence intervals around the predicted values. (B & D) Parameter estimates (mean of the posterior distribution) and 95% credible intervals (CI) for all fixed effects. Instances where the 95% CI overlaps zero are colored in purple.

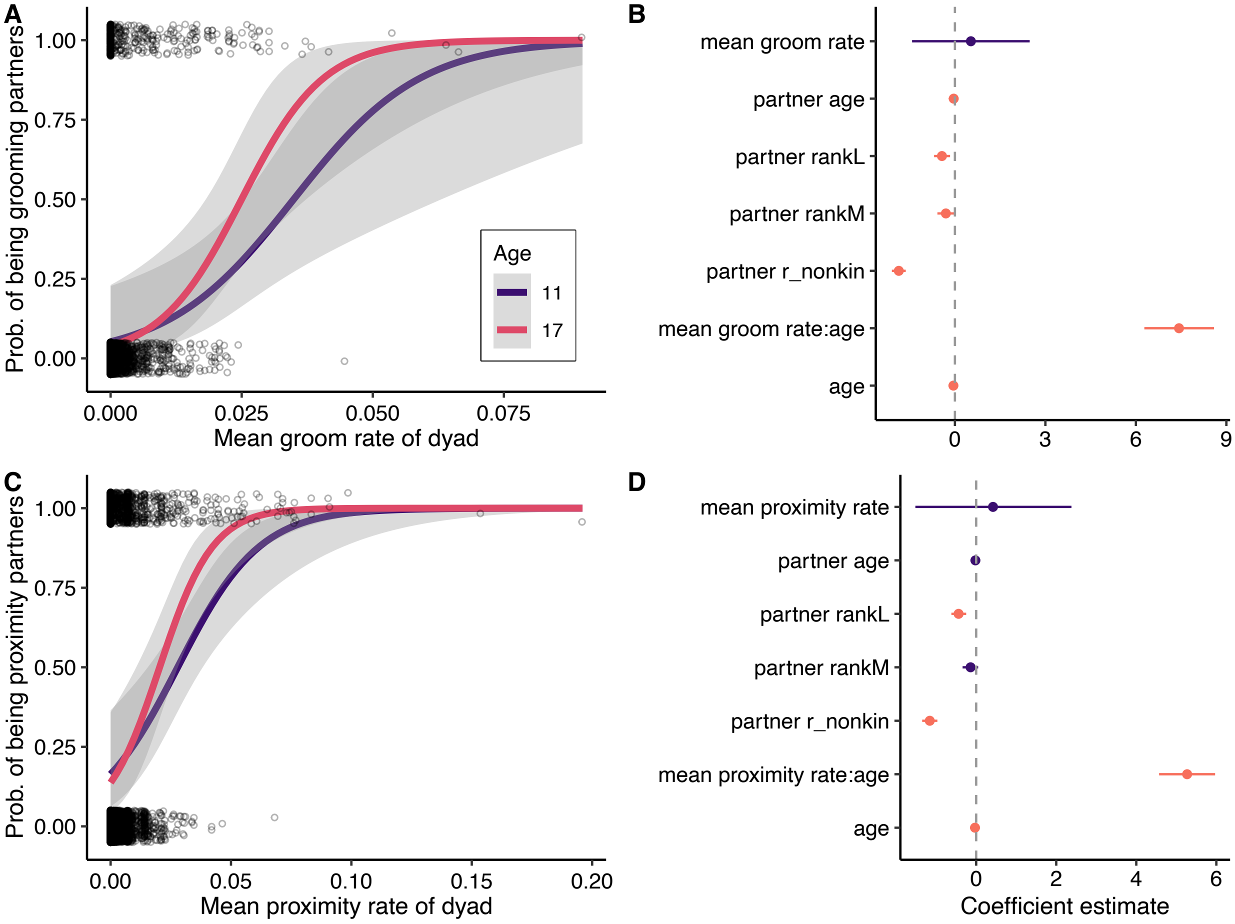

Table S15. Fixed and random effects from models looking at whether the relationship stability between a female and her grooming partner (i.e., being grooming partners for at least two consecutive years) predicts the probability of those individuals being grooming partners later in the female's life. Bolded terms indicate fixed effects where the 95% credible intervals did not overlap zero, providing evidence that those effects were significantly different from zero.

We ran the model with the following weakly informative prior means and standard deviations (μ, σ): intercept (0, 0.5), grooming partner stableY (0, 1), partner age (0, 1), partner rankL (0, 1), partner rankM (0, 1), partner relatedness_nonkin (0, 1), groupV (0, 1), groupKK (0, 1), grooming partner stableY:age (0, 1).

Effect	Group	Term	Estimate	Lower 95% CI	Upper 95% CI
**Fixed Effects**		intercept	2.57	1.12	4.00
		grooming partner stableY	0.92	-0.30	2.13
		**partner age**	**-0.04**	**-0.08**	**-0.01**
		partner rankL	-0.35	-0.73	0.03
		partner rankM	-0.28	-0.68	0.13
		**partner relatedness_nonkin**	**-1.81**	**-2.13**	**-1.49**
		groupV	-0.24	-0.81	0.33
		**groupKK**	**0.99**	**0.49**	**1.48**
		**grooming partner stableY:age**	**0.14**	**0.06**	**0.22**
		**age**	**-0.05**	**-0.10**	**-0.01**
**Random Effects**	Year	sd(intercept)	3.88	1.89	7.34
	Multi-membership	sd(intercept)	0.91	0.61	1.22

Table S16. Fixed and random effects from models looking at whether the relationship stability between a female and her proximity partner (i.e., being proximity partners for at least two consecutive years) predicts the probability of those individuals being proximity partners later in the female's life. Bolded terms indicate fixed effects where the 95% credible intervals did not overlap zero, providing evidence that those effects were significantly different from zero.

We ran the model with the following weakly informative prior means and standard deviations (μ, σ): intercept (0, 0.5), proximity partner stableY (0, 1), partner age (0, 1), partner rankL (0, 1), partner rankM (0, 1), partner relatedness_nonkin (0, 1), groupV (0, 1), groupKK (0, 1).

Effect	Group	Term	Estimate	Lower 95% CI	Upper 95% CI
**Fixed Effects**		intercept	0.77	-0.42	2.00
		**proximity partner stableY**	**1.38**	**1.12**	**1.64**
		partner age	-0.02	-0.05	0.00
		**partner rankL**	**-0.39**	**-0.66**	**-0.12**
		partner rankM	-0.07	-0.35	0.20
		**partner relatedness_nonkin**	**-1.55**	**-1.80**	**-1.31**
		groupV	-0.47	-0.97	0.02
		**groupKK**	**1.87**	**1.44**	**2.28**
**Random Effects**	Year	sd(intercept)	1.83	0.59	3.94
	Multi-membership	sd(intercept)	0.95	0.76	1.17

Figure S6. Effects of the stability of the (A & B) grooming relationship and (C & D) proximity relationship between a female and her partner on the probability of that individual being chosen as a grooming or proximity partner, respectively, by the female in later life. Points represent raw data. Error bars indicate 95% confidence intervals around the predicted values. (B & D) Parameter estimates (mean of the posterior distribution) and 95% credible intervals (CI) for all fixed effects. Instances where the 95% CI overlaps zero are colored in purple.

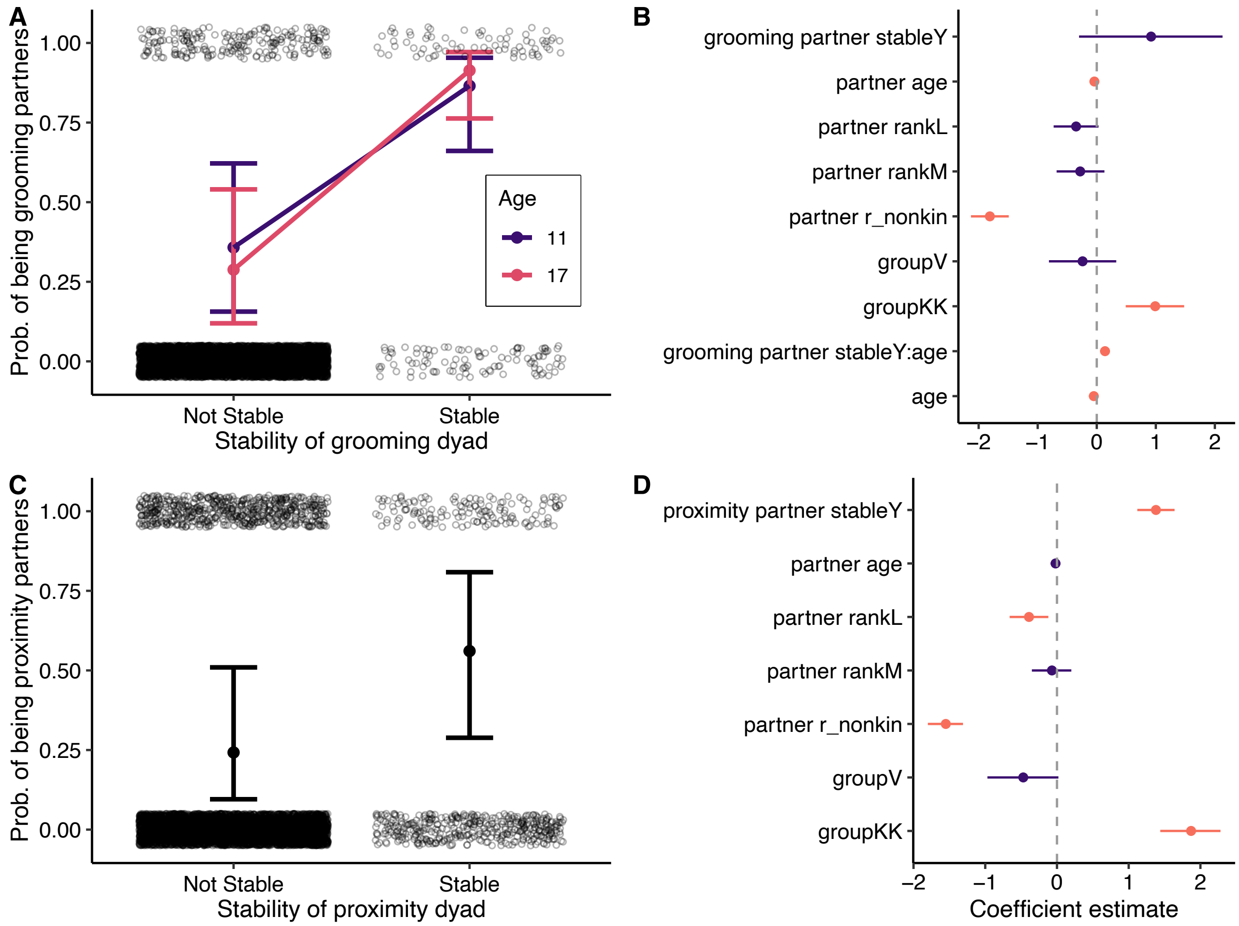

Table S17. Fixed and random effects from a post-hoc analysis looking at whether, at their last time point in the dataset (i.e., oldest age), females were more likely *to approach* partners with whom they had previously been strongly connected (i.e., had a high mean DSI). Bolded terms indicate fixed effects where the 95% credible intervals did not overlap zero, providing evidence that those effects were significantly different from zero.

We ran the model with the following weakly informative prior means and standard deviations (μ, σ): intercept (0, 0.5), mean DSI (0, 1), partner age (0, 1), partner rankL (0, 1), partner rankM (0, 1), partner relatedness_nonkin (0, 1).

Effect	Group	Term	Estimate	Lower 95% CI	Upper 95% CI
Fixed Effects		intercept	-0.99	-2.07	0.40
		**mean DSI**	**0.16**	**0.14**	**0.18**
		partner age	0.00	-0.02	0.02
		partner rankL	0.17	-0.02	0.36
		partner rankM	0.19	0.00	0.39
		**partner relatedness_nonkin**	**-0.97**	**-1.16**	**-0.78**
Random Effects	Group	sd(intercept)	0.91	0.34	2.37
	Year	sd(intercept)	1.16	0.56	2.45
	Multi-membership	sd(intercept)	0.90	0.76	1.05

Table S18. Fixed and random effects from a post-hoc analysis looking at whether, at their last time point in the dataset (i.e., oldest age), females were more likely *to be approached by* partners with whom they had previously been strongly connected (i.e., had a high mean DSI). Bolded terms indicate fixed effects where the 95% credible intervals did not overlap zero, providing evidence that those effects were significantly different from zero.

We ran the model with the following weakly informative prior means and standard deviations (μ, σ): intercept (0, 0.5), mean DSI (0, 1), partner age (0, 1), partner rankL (0, 1), partner rankM (0, 1), partner relatedness_nonkin (0, 1).

Effect	Group	Term	Estimate	Lower 95% CI	Upper 95% CI
**Fixed Effects**		intercept	0.82	-0.08	1.84
		**mean DSI**	**0.14**	**0.13**	**0.17**
		**partner age**	**-0.03**	**-0.04**	**-0.01**
		**partner rankL**	**-0.85**	**-1.03**	**-0.67**
		partner rankM	-0.10	-0.28	0.08
		**partner relatedness_nonkin**	**-0.97**	**-1.16**	**-0.79**
**Random Effects**	Group	sd(intercept)	1.23	0.43	3.00
	Year	sd(intercept)	0.92	0.42	2.02
	Multi-membership	sd(intercept)	0.75	0.62	0.88

Figure S7. Effect of social relationship strength (mean DSI) on the probability of (A) a female approaching or (C) being approached by a given individual in later life. Points represent raw data. Shaded gray ribbons indicate 95% confidence intervals around the predicted values. (B & D) Parameter estimates (mean of the posterior distribution) and 95% credible intervals (CI) for all fixed effects. Instances where the 95% CI overlaps zero are colored in purple.

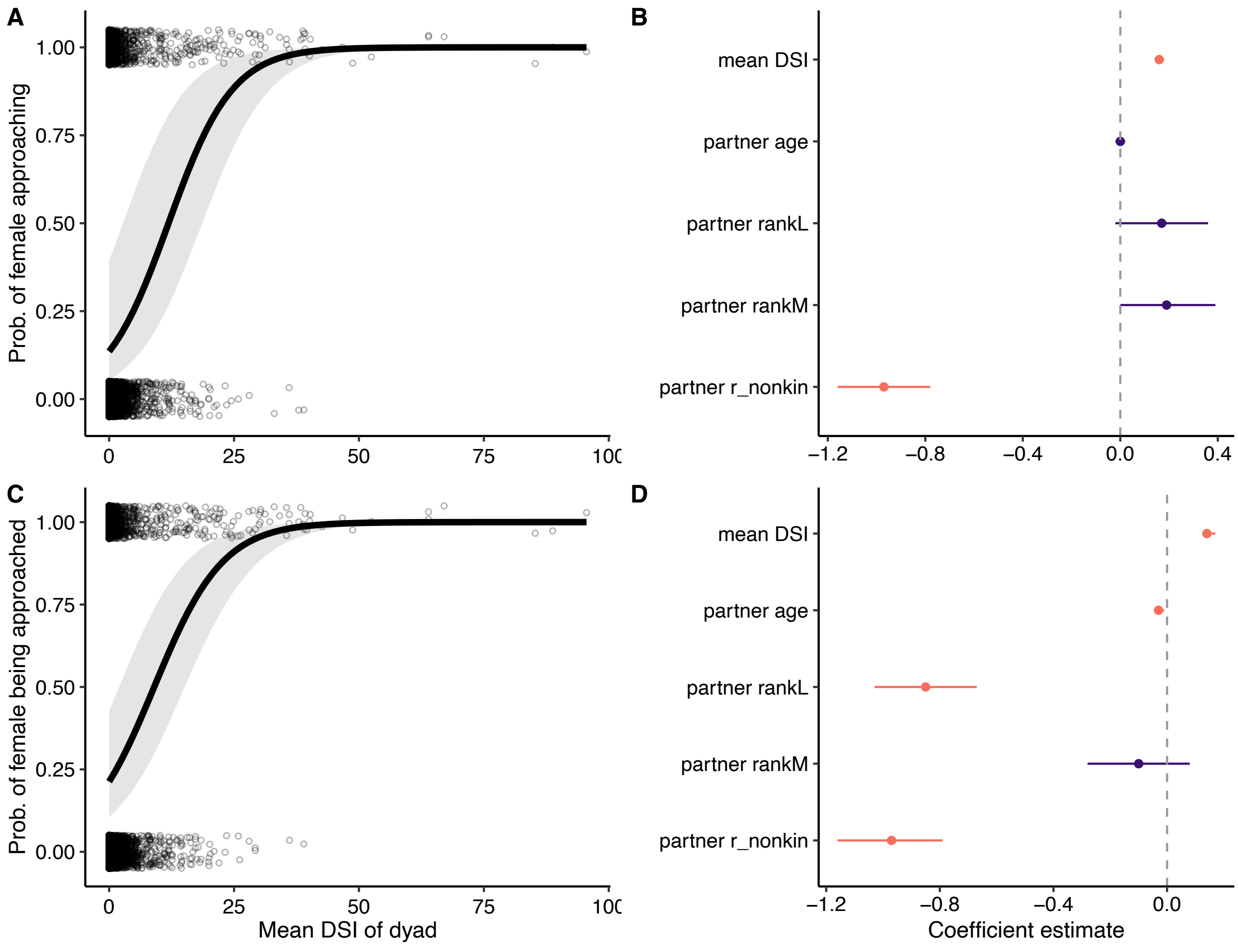

Table S19. Fixed and random effects from a post-hoc analysis looking at whether, at their last time point in the dataset (i.e., oldest age), females were more likely *to approach* partners with whom they had previously had a stable social relationship (i.e., been partners for at least two consecutive years). Bolded terms indicate fixed effects where the 95% credible intervals did not overlap zero, providing evidence that those effects were significantly different from zero.

We ran the model with the following weakly informative prior means and standard deviations (μ, σ): intercept (0, 0.5), partner stableY (0, 1), partner age (0, 1), partner rankL (0, 1), partner rankM (0, 1), partner relatedness_nonkin (0, 1), groupV (0, 1), groupKK (0, 1), partner stableY:age (0, 1).

Effect	Group	Term	Estimate	Lower 95% CI	Upper 95% CI
**Fixed Effects**		intercept	0.81	-0.5	2.12
		partner stableY	0.26	-0.52	1.03
		partner age	-0.01	-0.03	0.02
		**partner rankL**	**0.29**	**0.01**	**0.58**
		**partner rankM**	**0.35**	**0.06**	**0.63**
		**partner relatedness_nonkin**	**-1.41**	**-1.65**	**-1.17**
		groupV	-0.40	-0.88	0.07
		**groupKK**	**0.91**	**0.47**	**1.33**
		**partner stableY:age**	**0.06**	**0.02**	**0.11**
		age	-0.03	-0.07	0.00
**Random Effects**	Year	sd(intercept)	2.42	0.97	4.79
	Multi-membership	sd(intercept)	0.96	0.76	1.17

Table S20. Fixed and random effects from a post-hoc analysis looking at whether, at their last time point in the dataset (i.e., oldest age), females were more likely *to be approached by* partners with whom they had previously had a stable social relationship (i.e., been partners for at least two consecutive years). Bolded terms indicate fixed effects where the 95% credible intervals did not overlap zero, providing evidence that those effects were significantly different from zero.

We ran the model with the following weakly informative prior means and standard deviations (μ, σ): intercept (0, 0.5), partner stableY (0, 1), partner age (0, 1), partner rankL (0, 1), partner rankM (0, 1), partner relatedness_nonkin (0, 1), groupV (0, 1), groupKK (0, 1).

Effect	Group	Term	Estimate	Lower 95% CI	Upper 95% CI
**Fixed Effects**		intercept	0.69	-0.48	2.07
		**partner stableY**	**1.16**	**0.92**	**1.39**
		**partner age**	**-0.04**	**-0.06**	**-0.02**
		**partner rankL**	**-0.78**	**-1.02**	**-0.54**
		partner rankM	-0.22	-0.47	0.02
		**partner relatedness_nonkin**	**-1.24**	**-1.48**	**-1.01**
		groupV	-0.32	-0.73	0.08
		**groupKK**	**1.08**	**0.71**	**1.43**
**Random Effects**	Year	sd(intercept)	1.41	0.27	3.43
	Multi-membership	sd(intercept)	0.73	0.55	0.92

Figure S8. Effect of social relationship stability (i.e., being partners for at least two consecutive years) on the probability of (A) a female approaching or (C) being approached by a given individual in later life. Points represent raw data. Error bars indicate 95% confidence intervals around the predicted values. (B & D) Parameter estimates (mean of the posterior distribution) and 95% credible intervals (CI) for all fixed effects. Instances where the 95% CI overlaps zero are colored in purple.

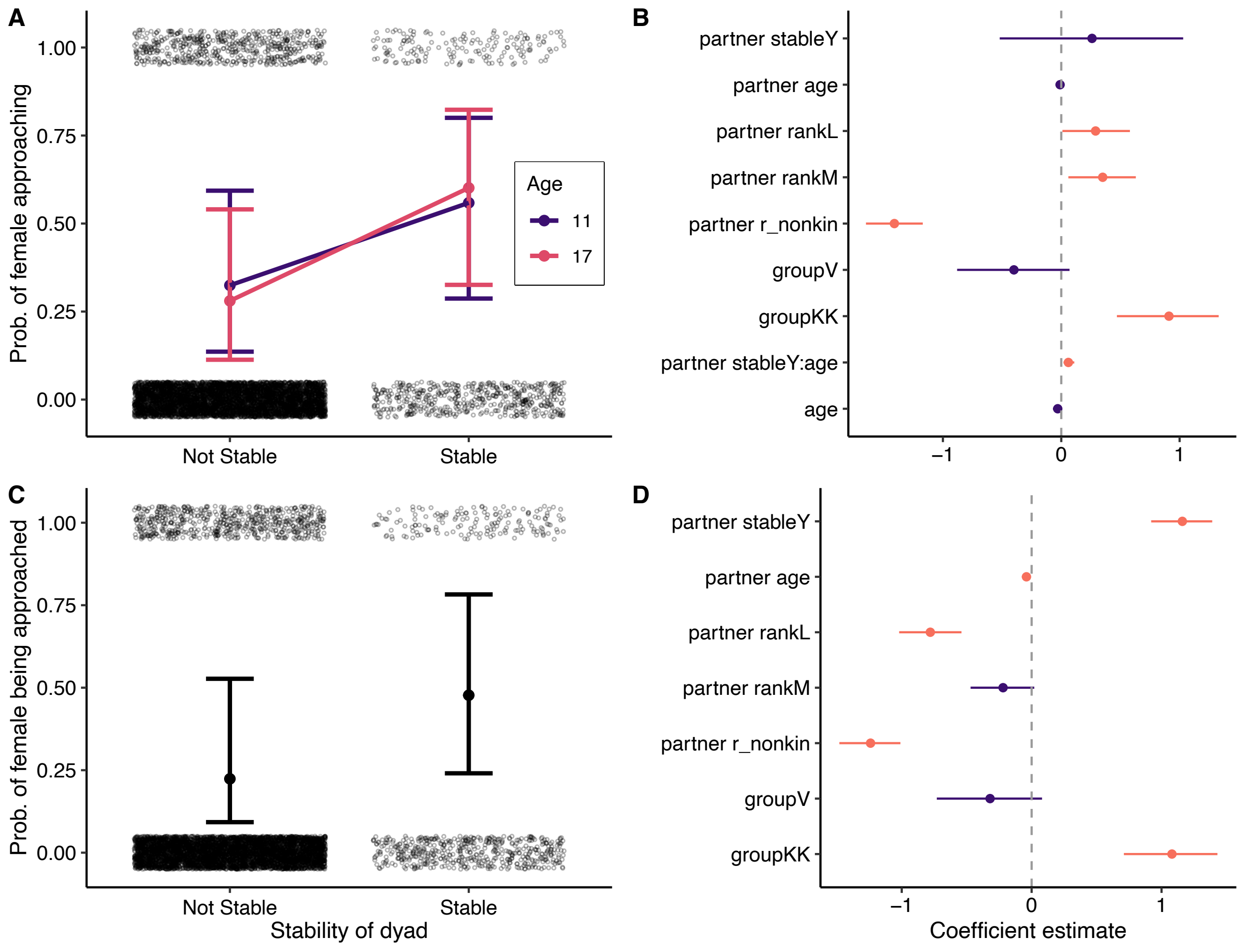

SI References

1. van de Pol M, Verhulst S (2006) Age-dependent traits: A new statistical model to separate within- and between-individual effects. *The American Naturalist* 167(5): 766–773.

2. van de Pol M, Wright J (2009) A simple method for distinguishing within- versus between-subject effects using mixed models. *Animal Behaviour* 77(3): 753–758.

3. Brent LJN, et al. (2013) Genetic origins of social networks in rhesus macaques. *Sci Rep* 3(1): 1361–8.

4. Brent LJN, Ruiz-Lambides A, Platt ML (2017) Family network size and survival across the lifespan of female macaques. *Proceedings of the Royal Society B: Biological Sciences* 284(1854): 20170515–7.

5. Ellis S, Snyder-Mackler N, Ruiz-Lambides A, Platt ML, Brent LJN (2019) Deconstructing sociality: the types of social connections that predict longevity in a group-living primate. *Proceedings of the Royal Society B: Biological Sciences* 286(1917): 20191991–10.

6. Tranmer M, Steel D, Browne WJ (2014) Multiple-membership multiple-classification models for social network and group dependences. *Journal of the Royal Statistical Society Series A (Statistics in Society)* 177(2): 439–455.
